# SMURF Reconstructs Single-Cells from Visium HD Data to Reveal Zonation of Transcriptional Programs in the Intestine

**DOI:** 10.1101/2025.05.28.656357

**Published:** 2025-06-01

**Authors:** Juanru Guo, Simona Sarafinovska, Ryan A. Hagenson, Mark C. Valentine, Joseph D. Dougherty, Robi D. Mitra, Brian D. Muegge

## Abstract

Spatial transcriptomics (ST) promises to reveal the principles of tissue organization and regional variation. With the advent of high-resolution capture-based ST methods such as Visium HD, new approaches are needed that faithfully assign transcripts to individual cells and enable the analysis of cells in complex tissue substructures. Here we introduce the Segmentation and Manifold UnRolling Framework (SMURF), a computational tool for the analysis of high-resolution ST data. SMURF introduces a novel “soft-segmentation” approach that accurately aggregates and assigns the mRNAs from capture spots to nearby cells. SMURF is also able to “unroll” cells contained in complex tissue structures and place them on standard Cartesian coordinates, thus empowering studies of cellular organization and regional specification. We demonstrate the power of SMURF in the mouse small intestine. We identified the zonation of metabolic programs along the maturing intestinal villus and the transcription factors that regulate them. We used SMURF to investigate an important outstanding question – how are functionally important proximal-to-distal gene expression gradients in the intestine specified. We discovered that regionally specific gene programs along the proximal-distal axis accumulate in the villus tip and that the tip is reprogrammed by environmental signals in the lumen, suggesting these inputs are a major determinant of regional transcriptional identity. SMURF promises to accelerate the effective use of high-resolution ST data by enabling more accurate segmentation and assignment of mRNAs to cells and by providing a unique toolbox for the analysis of regional transcriptional programs along tissue manifolds.

## Full Text Availability

The license terms selected by the author(s) for this preprint version do not permit archiving in PMC. The full text is available from the preprint server.

